# Reiter`s syndrome following *Salmonella* infection

**DOI:** 10.1186/1471-2334-14-S7-P58

**Published:** 2014-10-15

**Authors:** Mirela Indrieş

**Affiliations:** 1 Municipal Clinical Hospital "Dr. Gabriel Curteanu" Oradea, Romania

## Background

Reactive arthritis, Reiter's syndrome is one of the seronegative arthropathies, that can be associated with intestinal infections (*Shigella*, *Salmonella*, *Yersinia*, *Campylobacter jejuni*, *Clostridium difficile*), sexual infections (*Chlamydia trachomatis*, *Ureaplasma urealyticum*) and lung infections (*Chlamydia pneumoniae*, *Mycoplasma pneumoniae*). Reiter's syndrome is an arthritis that occurs 1-4 weeks in response to an infection with a specific organism with urogenital or enteral gate, especially in HLA-B27 positive individuals.

## Case report

We present the case of a patient of 34 years, from a family outbreak of food-borne *Salmonella* infection, presented 10 days after discharge from the Department of Infectious Diseases Oradea after treatment with ampicillin 4 g/day and ciprofloxacin 1 g/day, with a fever, swelling of the right ankle and left knee, accompanied by secondary functional impotence at this level and conjunctivitis. Biological: inflammatory syndrome, minimum elevated liver enzymes, negative rheumatoid factor, normal joint radiography, HLA B 27 positive; abdominal CT showed retroperitoneal inflammatory lymphadenopathy. Under antibiotic therapy, corticosteroid, anti-inflammatory and hepatoprotectives, evolution was undulating, with three episodes of relapse within 6 months.

## Conclusion

Antibiotic treatment during both acute infection and Reiter’s syndrome shortened the evolution period in this case.

## Consent

Written informed consent was obtained from the patient for publication of this Case report and any accompanying images. A copy of the written consent is available for review by the Editor of this journal.

